# *Bacillus amyloliquefaciens* TL promotes gut health of broilers by the contribution of bacterial extracellular polysaccharides through its anti-inflammatory potential

**DOI:** 10.3389/fimmu.2024.1455996

**Published:** 2024-09-23

**Authors:** Shijie Li, Pinpin Chen, Qiuyuan Li, Xu Wang, Jintao Peng, Ping Xu, Hongxia Ding, Zutao Zhou, Deshi Shi, Yuncai Xiao

**Affiliations:** ^1^ National Key Laboratory of Agricultural Microbiology, Huazhong Agricultural University, Wuhan, China; ^2^ College of Veterinary Medicine, Huazhong Agricultural University, Wuhan, China; ^3^ Key Laboratory of Preventive Veterinary Medicine in Hubei Province, Huazhong Agricultural University, Wuhan, China

**Keywords:** *Bacillus amyloliquefaciens* TL, broiler intestine, inflammatory factors, extracellular polysaccharides, anti-inflammatory activity, NF-κB signaling pathway

## Abstract

The focal point of probiotic efficacy and a crucial factor influencing poultry cultivation lies in the level of intestinal inflammation. In conventional farming processes, the reduction of intestinal inflammation generally proves advantageous for poultry growth. This study investigated the impact of *Bacillus amyloliquefaciens* TL (B.A.-TL) on inflammatory factor expression at both tissue and cellular levels, alongside an exploration of main active secondary metabolites. The results demonstrated that broiler feeding with a basal diet containing 4 × 10^9^ CFU/kg B.A.-TL markedly enhanced chicken growth performance, concomitant with a significant decrease in the expression of genes encoding inflammatory cytokines (e.g., *CCL4*, *CCR5*, *XCL1*, *IL-1β*, *IL-6*, *IL-8*, *LITAF*, and *LYZ*) in jejunum and ileum tissues. The extracellular polysaccharides of B.A.-TL (EPS-TL) exhibited notable suppression of elevated inflammatory cytokine expression induced by *Escherichia coli* O55 lipopolysaccharides (LPS) in chicken macrophage-like cells (HD11) and primary chicken embryonic small intestinal epithelial cells (PCIECs). Moreover, EPS-TL demonstrated inhibitory effect on NF-κB signaling pathway activation. These findings suggested that the metabolic product of B.A.-TL (i.e., EPS-TL) could partly mitigate the enhanced expression of inflammatory factors induced by LPS stimulation, indicating its potential as a key component contributing to the anti-inflammatory effects of B.A.-TL.

## 1 Introduction

*Bacillus amyloliquefaciens*, characterized as a motile Gram-positive, aerobic, and rod-shaped bacterium, exhibits widespread distribution across various environments, encompassing air, soil, freshwater, marine ecosystem, plant, and animal body, reflecting considerable geographical and host diversity. Acknowledged as a Grade A safe strain in the “General Technical Guidelines for the Biosafety of Microbial Fertilizers” by the Chinese Ministry of Agriculture, *B. amyloliquefaciens* stands among the first group of bacterial strains certified as safe by the FDA in the USA. The majority of *B. amyloliquefaciens* strains are non-toxic and environmentally benign, causing no risks of water source contamination. In recent years, *B. amyloliquefaciens* has garnered significant attention and application in livestock and poultry farming. Numerous reports have demonstrated that *B. amyloliquefaciens* typically exerts favorable effects on the production performance of white feather broilers, i.e., enhancing broiler growth rates (1–4), fostering intestinal development, improving tissue morphology (5), modulating gut microbial structure (1, 6), and augmenting short-chain fatty acids, digestive enzymes, and total antioxidants (7), thereby significantly improving feed conversion rates. *Bacillus amyloliquefaciens* also enhances the growth performance of laying hens, resulting in a substantial increase in egg production and high-quality egg rates (8). Furthermore, this bacterium effectively enhances rooster sperm quality, chicken embryo hatchability (8, 9), and may even moderately delay the reproductive aging of laying hen ovaries (6, 10). Additionally, *B. amyloliquefaciens* appears to positively influence avian serum biochemical indicators, organ immune cytokines, and immune cell functions (1, 11, 12).

Inflammation functions as the body’s primary adaptive defense mechanism in response to damage or pathology, permeating various biological processes and facilitating the organism’s removal of anomalies and lesions. In poultry, primary inflammatory factors encompass chemokines, interleukins, enzymes, antimicrobial peptides, and other bioactive substances (13–15). In high-density poultry farming, the digestive tracts and respiratory system practically emerge as significant sites of morbidity. Within the intestinal tissues, diseases and stressors can incite varying degrees of inflammation, with the intensity of the inflammatory response closely linked to the stage and severity of the ailment or stressor. For example, in disease models induced by bacteria or lipopolysaccharides (LPS) (16–18), viruses (19, 20), parasites (21, 22) or heat stress (23, 24) in broilers, pathogens rapidly initiate intestinal or localized inflammations, which often lead to increased expression of pro-inflammatory factors such as IL-1β, IL-6, and IL-8, as well as intestinal damage and functional impairment. As a result, elevated intestinal inflammation generally exerts a detrimental impact on poultry production. Numerous studies affirm that poultry exhibiting superior growth or egg-laying performance typically manifest low levels of intestinal inflammation (25–27). Effective mitigation of the onset and progression of inflammation in poultry pathological models frequently proves advantageous for the production performance (16, 28). It is apparent that, whether under physiological or pathological conditions, the growth and intestinal inflammation in broilers demonstrate a highly correlated relationship. Notably, reduced inflammatory responses generally foster enhanced growth performance in broilers, a proposition strongly substantiated by extant evidence.

In recent years, microbe-derived exopolysaccharides (EPS) have increasingly garnered scientific attention in the realms of animal and plant research due to their beneficial effects and potential applications. The biological activities of bacterial EPS, particularly their antioxidant properties, have been a focal point of investigation. Various sources of EPS could effectively scavenge –OH and O_2_^–^, augmenting the content of antioxidant enzymes in body (29–31). Moreover, certain proportion of EPS exhibits anti-tumor properties, effectively mitigating complications associated with malignant tumors while attenuating inflammatory damage (32, 33). Additionally, various types of EPS have been scrutinized for their potential in reducing blood sugar and lipids, anti-aging effects, inhibition of biofilm formation, as well as antimicrobial and antiviral activities (34–36). Notably, specific bacterial EPS display immunomodulatory activities affecting the inflammatory response and immune regulation of animals. Research has demonstrated that *lactic acid bacterial* EPS significantly inhibit the activation of intestinal inflammation pathways and effectively reduce the inflammatory response of intestinal epithelial cells (37). At the cellular level, these EPS augment the phagocytic activity of macrophages while inhibiting the overexpression of inflammatory cytokines (38), showcasing robust immunomodulatory potential. Furthermore, certain bacterial EPS can enhance the immune function of animal intestines (39) and positively modulate the structure of intestinal microbial community (40). It is apparent that bacterial EPS encompass numerous potent biological activities, underscoring their potential as feed additives. To date, the biological characteristics of EPS generated by *B. amyloliquefaciens* remain under investigated.

## 2 Materials and methods

### 2.1 Bacterial strain, cell line, plasmid, and antibodies

Strain B.A.-TL (patent number ZL 2015 1 0551645.9) was maintained at the Laboratory of Veterinary Microbiology and Immunology, College of Veterinary Medicine, Huazhong Agricultural University (Wuhan, China). The microecological preparations of B.A.-TL were provided by Hubei Huada Real Technology Co., Ltd. The chicken macrophage-like cell line HD11 was preserved in the Laboratory of Animal Physiology, College of Veterinary Medicine, Huazhong Agricultural University. Primary chicken embryonic small intestinal epithelial cells (PCIECs) were isolated (41, 42) from 18- and 19-day-old Leghorn specific pathogen-free chicken embryos (Beijing Boehringer Ingelheim Vital Biotechnology Co., Ltd., Beijing, China). The plasmid (pEN-mCherry-Linker) was purchased from Miaoling Biotechnology Co., Ltd. (Wuhan, China). Rabbit anti-chicken NF-κB P65 antibody (1:900) was obtained from Bioss Biotechnology Co., Ltd. (Cat. bs-0465R; Beijing, China), rabbit anti-chicken GAPDH antibody (1:20000) was obtained from Huabio Biotechnology Co., Ltd. (Cat. ET1702-66; Hangzhou, China), rabbit anti-IκBα antibody (1:800) was obtained from Cell Signaling Technology Co., Ltd. (Cat. 9242S; MA, USA), and horseradish peroxidase-conjugated (HRP) goat anti-rabbit (1:10000) antibody was obtained from ABClonal Biotechnology Co., Ltd. (Cat. AS014; Wuhan, China).

### 2.2 Extraction and analysis of fermentation supernatant, extracellular polysaccharides, and proteins from *Bacillus amyloliquefaciens* TL

The B.A.-TL fermentation supernatant analytical procedure was provided in **Supplementary Materials and methods 1.1**.

The B.A.-TL fermentation supernatant was subjected to a water bath at 100°C for 30 min, followed by cooling and filtration to eliminate bacterial cells. Anhydrous ethanol was added in a volume triple that of the supernatant, and the mixture was allowed to precipitate at 4°C overnight. Then, the precipitate was collected by centrifugation at 8000 rpm for 5 min; after that the supernatant was discarded, and the pellet was resuspended in sterile distilled water to obtain a crude polysaccharide solution. Sevage reagent (chloroform: n-butanol = 4:1; V/V) was added at 0.2 times the volume for deproteinization. After stirring for 30 min, the mixture underwent centrifugation to eliminate denatured protein components. This process was iterated until no protein precipitation observed. The solution underwent dialysis in PBS (molecular weight cutoff of 3 kDa) for 48–72 h. The dialysate was freeze-dried and stored at –80°C. 1 mg sample of B.A.-TL extracellular polysaccharides (EPS-TL) was dissolved in 1 mL distilled water to prepare the test sample (43). Both test sample and standard solutions were subjected to analysis for monosaccharide composition utilizing the Thermo ICS5000 ion chromatography system (Thermo Fisher Biotechnology Co., Ltd., MA, USA) with a Dionex™ CarboPac™ PA20 column (150 × 3.0 mm, 10 μm).

The B.A.-TL fermentation supernatant was centrifuged at 8000 rpm to eliminate bacterial cells. While stirring on ice, ammonium sulfate powder was gradually added to the supernatant until 90% saturation was achieved. The mixture was gently stirred for 6–12 h and then allowed to precipitate proteins overnight at 4°C. The supernatant was discarded, and crude protein was collected by centrifugation at 12000 rpm for 30 min. After discarding the supernatant, the pellet was resuspended in sterile distilled water, followed by dialysis (until no SO_4_^2-^ was detected in the dialysate). The total extracellular proteins (EPro-TL) of B.A.-TL were then mixed with glycerol in a 1:1 (V/V) ratio and stored at –20°C (44). Protein concentration was determined using the bicinchoninic acid Protein Quantification Kit (Epizyme Biotechnology Co., Ltd., Shanghai, China).

### 2.3 Experimental animals

A total of 120 male Cobb500 broilers were procured for the study (43, 45) and randomly allocated into a B.A.-TL group (fed with a basic diet containing 4 × 10^9^ CFU/kg B.A.-TL) and a control group (fed with basic diet). The formulation and nutritional components of the broiler pellet feed were outlined in **Supplementary Table 1**. Both B.A.-TL and control groups comprised 6 replicates, with 10 chicks per replicate individually tagged using leg bands. The broilers received regular feeding and their raising condition was provided in **Supplementary Materials and methods 1.2**.

### 2.4 Tissue sampling and sectioning

Each group of samples had no less than 8 replicates. The sampling and sectioning methods were provided in **Supplementary Materials and methods 1.3**.

### 2.5 RNA extraction, cDNA library preparation and quantitative real-time PCR

The methods of RNA extraction, cDNA library preparation and quantitative real-time PCR (qRT-PCR) were provided in **Supplementary Materials and methods 1.4**.

### 2.6 Illumina sequencing

The sequencing, quality control and analysis were performed via the second generation high-throughput platform (Majorbio Biotechnology Co., Ltd., Shanghai, China), the related methods were provided in **Supplementary Materials and methods 1.5**.

### 2.7 CCK-8 cell proliferation assay

The experimental groups were established as follows: the treatment group contained HD11 cells, B.A.-TL cells, fermentation supernatant, EPS-TL, and EPro-TL; the blank control group contained HD11 cells and DMEM basal culture medium (Gibco Biotechnology Co., Ltd., MA, USA); the positive control group included HD11 cells, DMEM basal culture medium, and 10% FBS (Gibco Biotechnology Co., Ltd., MA, USA); and the negative control group contained only DMEM basal culture medium. Each group consisted of 6 biological replicates. HD11 cells were seeded in a 96-well plate at a density of 5 × 10^3^ cells/well and allowed to reach 60–70% confluence. After washing three times with PBS, different stimuli were sequentially added, with concentrations and treatment durations determined based on a concentration-time gradient. After the stimulation, cells were washed with PBS, and 10 μL of CCK-8 colorimetric reagent (GlpBio Biotechnology Co., Ltd., CA, USA) was added to each well. The system was then incubated for 1–2 h to facilitate color development (43). Following the incubation, absorbance at 450 nm was measured using a MK3 microplate reader (Thermo Fisher Biotechnology Co., Ltd., MA, USA).

### 2.8 mRNA expression analysis of inflammatory factors

HD11 cells underwent stimulation with a series of dilutions of B.A.-TL supernatant, and different concentrations of B.A.-TL cells, EPS-TL, and EPro-TL, as well as LPS of *Escherichia coli* O55 (Sigma Biotechnology Co., Ltd., MO, USA), using a concentration-time gradient procedure to perform pre-treatment, simultaneous treatment, and post-treatment, respectively. Stimulation was carried out with varied dilution factors and concentrations to establish a concentration-time gradient. Following treatment, HD11 cells were washed three times with PBS. Subsequently, Trizol reagent (BioSharp Biotechnology Co., Ltd., Hefei, China) was employed to lyse the cells and collect the samples. RNA extraction, cDNA library preparation and qRT-PCR were conducted as described in Section 2.5. Primers were provided in **Supplementary Table 2**.

### 2.9 ELISA analysis of inflammatory factors

HD11 cells were first treated as described in Section 2.8. ELISA kits (MEIMIAN Biotechnology Co., Ltd., Yancheng, China; CCL4, Cat. MM-60080O2; IL-1β, Cat. MM-36910O2; IL-8, Cat. MM-0768O2; LITAF, Cat. MM-0938O2) were used to perform the quantitative detection of inflammatory factors in the cell supernatant. The procedures of ELISA followed the manufacturer’s instructions.

### 2.10 Fluorescent and Western blot detection of cellular factors in NF-κB signaling pathway

The cDNA from HD11 cells was used as a template to amplify the P65 nuclear factor gene fragment using PrimeSTAR^®^ HS DNA Polymerase (GC Buffer) high-fidelity enzyme (Takara Biotechnology Co., Ltd., Beijing, China). Primers were provided in **Supplementary Table 2**. The pEN-mCherry-P65 eukaryotic expression vector was constructed (**Supplementary Figure 1**). HD11 cells were seeded in a 6-well plate, and when the cell confluence reached approximately 70%, the plasmid was transfected into HD11 cells using Lipofectamine 2000 (Thermo Fisher Biotechnology Co., Ltd., MA, USA). Subsequently, the cells were treated as described in Section 2.8. After treatment, cells were washed three times with PBS, fixed in 4% paraformaldehyde, and added with the anti-fluorescence quenching agent (Beyotime Biotechnology Co., Ltd., Shanghai, China). The cells were then stored in the dark at 4°C. The localization of nuclear factor P65 (indicated by mCherry fluorescence) in HD11 cells was captured using the LSM 900 laser confocal imaging system (Zeiss Co., Ltd., Oberkochen, Germany) with software ZEN 2.3 (blue edition), which facilitated the visualization of P65 distribution within the nucleus and cytoplasm.

HD11 cells were treated as described in Section 2.8. Following treatment, HD11 cells were washed three times with PBS, either added with RIPA lysis buffer (Beyotime Biotechnology Co., Ltd., Shanghai, China) containing a protease inhibitor or repeatedly freeze-thawed multiple times with metal ion-free DEPC buffer (Beyotime Biotechnology Co., Ltd., Shanghai, China), and then incubated on ice. The cell lysates were subjected to separation by 10% sodium dodecyl sulfate-polyacrylamide gel (containing 5 mM Phos-tag™ Acrylamide AAL-107 0.09 mL and 10 mM MnCl_2_ solution 0.09 mL under experimental requirement; Fujifilm WAKO Biotechnology Co., Ltd., Hongkong, China) electrophoresis (SDS-PAGE) (46, 47). Subsequently, the proteins were transferred to a polyvinylidene difluoride (PVDF) membrane (Bio-Rad Biotechnology Co., Ltd., CA, USA), which was blocked with TBST blocking solution (BD Biotechnology Co., Ltd., CA, USA) containing either 5% bovine serum albumin (BSA; Thermo Fisher Biotechnology Co., Ltd., MA, USA) or 5% skim milk (BD Biotechnology Co., Ltd., CA, USA), under different experimental protocols for 2 h (48), followed by overnight incubation at 4°C with the primary antibodies. Afterward, the membrane was incubated for 1 h with the appropriate HRP-conjugated secondary antibodies. Proteins transferred were exposed and imaged in gel imaging system (Bio-Rad Biotechnology Co., Ltd., CA, USA). Quantitative analysis of protein bands was conducted using software Image J 1.53c.

### 2.11 Statistical analysis

The experimental data were analyzed using software IBM SPSS Statistics 25, and data visualization was performed using software GraphPad Prism 8. Significance analysis of differences between sample groups was conducted using Student t-tests and one-way ANOVA based on *p* < 0.05 (*), *p* < 0.01 (**), *p* < 0.001 (***), and *p* < 0.0001 (****), respectively.

## 3 Results

### 3.1 Growth performance of broiler chickens

The average body weight, average daily gain, and feed conversion ratio (FCR) of Cobb500 broilers were presented in **Table 1**. Notably, B.A.-TL group displayed a significantly higher final body weight compared to control group (*p* < 0.05), with the more pronounced disparity detected during the first 3 weeks (*p* < 0.01). Moreover, B.A.-TL group exhibited a higher weekly weight gain relative to control group, especially during the first 3 weeks, and the weekly weight gain rate surpassed that of control group, albeit experiencing a slight decrease in the fourth and fifth weeks compared to control group. The FCR averaged 1.58 for control group over the 5-week period, whereas B.A.-TL group demonstrated an FCR of 1.50, indicating a relative decrease of 4.95%.

**Table 1:** Effect of *Bacillus amyloliquefaciens* TL on the growth performance in Cobb500 broiler chickens.

Item and time	Treatment		*p*-value
	Control group	B.A.-TL group	
Average body weight (g)			
Initial	63.29 ± 2.48	63.23 ± 2.88	0.9097
7 d	176.77 ± 19.07^B^	188.09 ± 18.69^A^	0.0019
14 d	367.68 ± 52.40^B^	407.33 ± 48.14^A^	0.0000
21 d	784.79 ± 125.99^B^	867.89 ± 134.39^A^	0.0093
28 d	1290.16 ± 166.15^b^	1425.28 ± 194.94^a^	0.0249
35 d	1934.90 ± 205.21^b^	2122.21 ± 223.48^a^	0.0167
Average daily gain (g) and weight gain rate (%)			
Week 1	110.47 (1.75%)	119.83 (1.90%)	
Week 2	190.8 (1.08%)	217.69 (1.15%)	
Week 3	420.36 (1.14%)	467.17 (1.15%)	
Week 4	469.75 (0.60%)	482.63 (0.56%)	
Week 5	622.03 (0.48%)	661.98 (0.46%)	
Food conversion ratio (1–35 d)			
Total	1.58 ± 0.20	1.50 ± 0.18	0.5343

Data are expressed as “mean ± standard error of the mean” (60 chickens counted in each group). Means within the same row with different superscripts differ significantly at *p* < 0.01 (^A^ and ^B^) and *p* < 0.05 (^a^ and ^b^), respectively.

### 3.2 Intestinal tissue sectioning and staining

**Figure 1:**
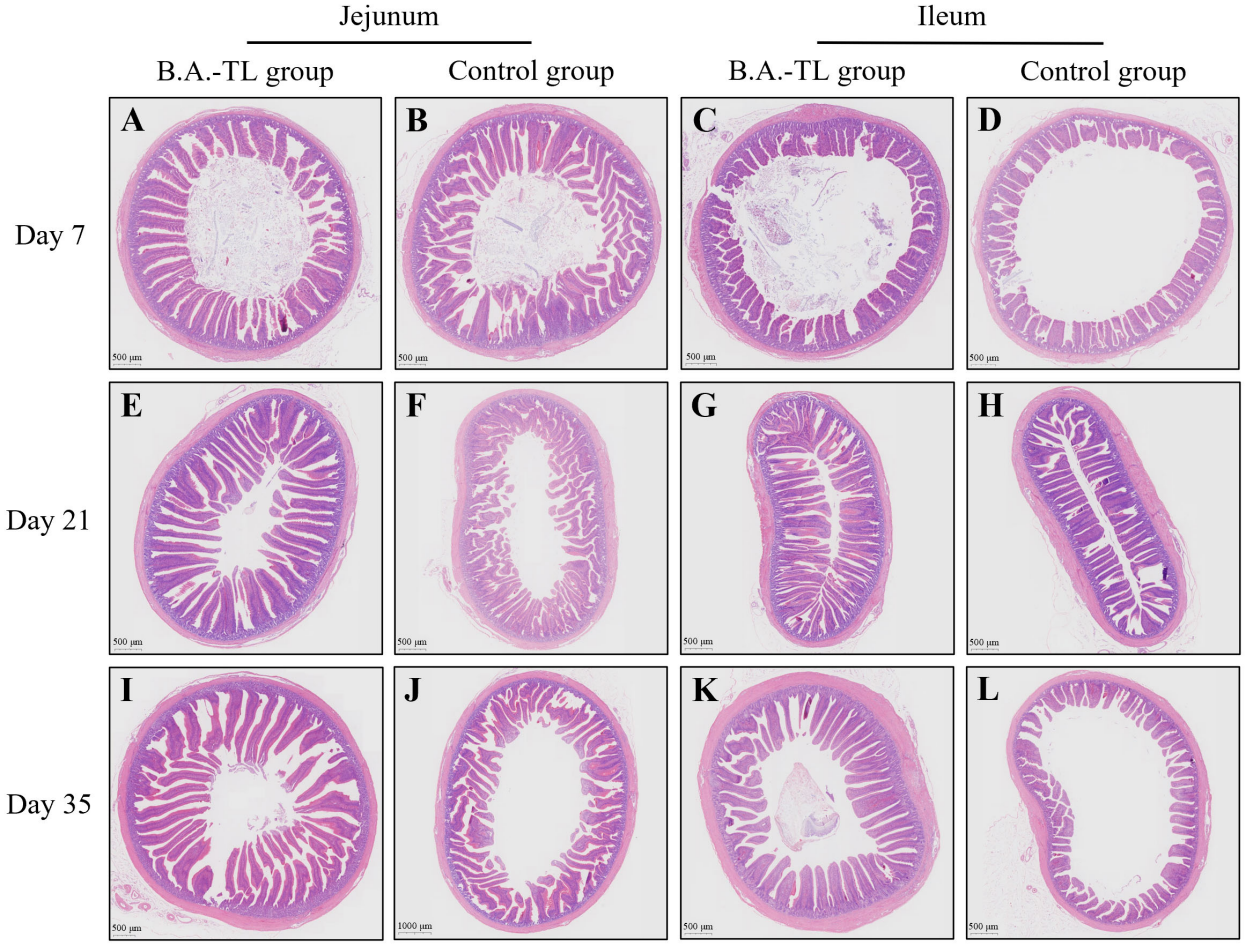
H&E staining of intestinal tissue sections of broiler chickens in both B.A.-TL and control groups at days 7 **(A–D)**, 21 **(E–H)**, and 35 **(I–L)**, respectively.

Histological sections of the jejunum and ileum tissues from Cobb500 broilers were examined (**Figure 1**). Software CaseViewer 2.4 RTM was used to collect at least 5 random measurements of villus height (Vh) and crypt depth (Cd) for each group, with the Vh/Cd ratio calculated (VCR; **Table 2**). The jejunum and ileum Vh of chickens in B.A.-TL group were significantly higher than those of control group at 7, 21, and 35 d of age (*p* < 0.05). Both the jejunum VCR (*p* < 0.05) and ileum VCR (*p* < 0.01) were significantly higher in B.A.-TL group compared to control group. No significant difference was observed in Cd between the two groups during the entire experiment.

**Table 2:** Intestinal villus height and crypt depth analysis in broiler chickens.

Character	Control group		B.A.-TL group		*p*-value
	Jejunum	Ileum	Jejunum	Ileum	
Day 7					
Villus height (μm)	823.42 ± 24.17^b^	388.51 ± 24.80^d^	952.26 ± 46.85^a^	494.23 ± 23.24^c^	< 0.05
Crypt depth (μm)	117.84 ± 8.07	116.52 ± 6.40	124.34 ± 3.73	118.66 ± 9.36	0.79
VCR	6.99 ± 0.46^b^	3.27 ± 0.39^d^	7.65 ± 0.41^a^	4.24 ± 0.35^c^	< 0.05
Day 21					
Villus height (μm)	1042.81 ± 28.57^b^	468.89 ± 97.02^D^	1139.89 ± 32.92^a^	566.95 ± 22.80^C^	< 0.01
Crypt depth (μm)	157.22 ± 14.04	123.73 ± 10.06	153.25 ± 10.94	123.95 ± 15.69	0.82
VCR	6.63 ± 0.65^B^	3.79 ± 0.96^D^	7.44 ± 0.36^A^	4.57 ± 0.70^C^	< 0.01
Day 35					
Villus height (μm)	1428.40 ± 28.44^b^	526.17 ± 27.46^D^	1551.40 ± 98.32^a^	684.21 ± 18.32^C^	< 0.01
Crypt depth (μm)	187.03 ± 20.37	144.84 ± 12.15	190.37 ± 14.03	154.23 ± 12.36	0.67
VCR	7.64 ± 0.70^b^	3.63 ± 0.16^d^	8.15 ± 0.89^a^	4.43 ± 0.32^c^	< 0.05

Data are expressed as “mean ± standard error of the mean” (5 replicates of villus height and crypt depth counted in each group). VCR, the villus height/crypt depth ratio. The significant difference is detected in the jejunal and ileal tissues of different groups at the same age, with each sample containing 5 replicates; means within a row with different superscripts differ significantly at *p* < 0.01 (^A^, ^B^, ^C^ and ^D^) and *p* < 0.05 (^a^, ^b^, ^c^ and ^d^), respectively.

### 3.3 Transcriptome of broiler chicken intestinal tissues

Transcriptome sequencing was performed on a total of 6 jejunum tissue (J) samples at day 21 (T/C_21_J_1–6_) and at day 35 (T/C_35_J_1-6_), and a total of 6 ileum tissue (I) samples at day 21 (T/C_21_I_1–6_) and at day 35 (T/C_35_I_1–6_) in both B.A.-TL (T) and control (C) groups of Cobb500 broilers. The sequencing data were aligned with the chicken reference genome (*Gallus gallus*; GCF_016700215.1), revealing an average overall alignment rate of 88.10%, with an average unique alignment rate of 85.37%.

**Figure 2:**
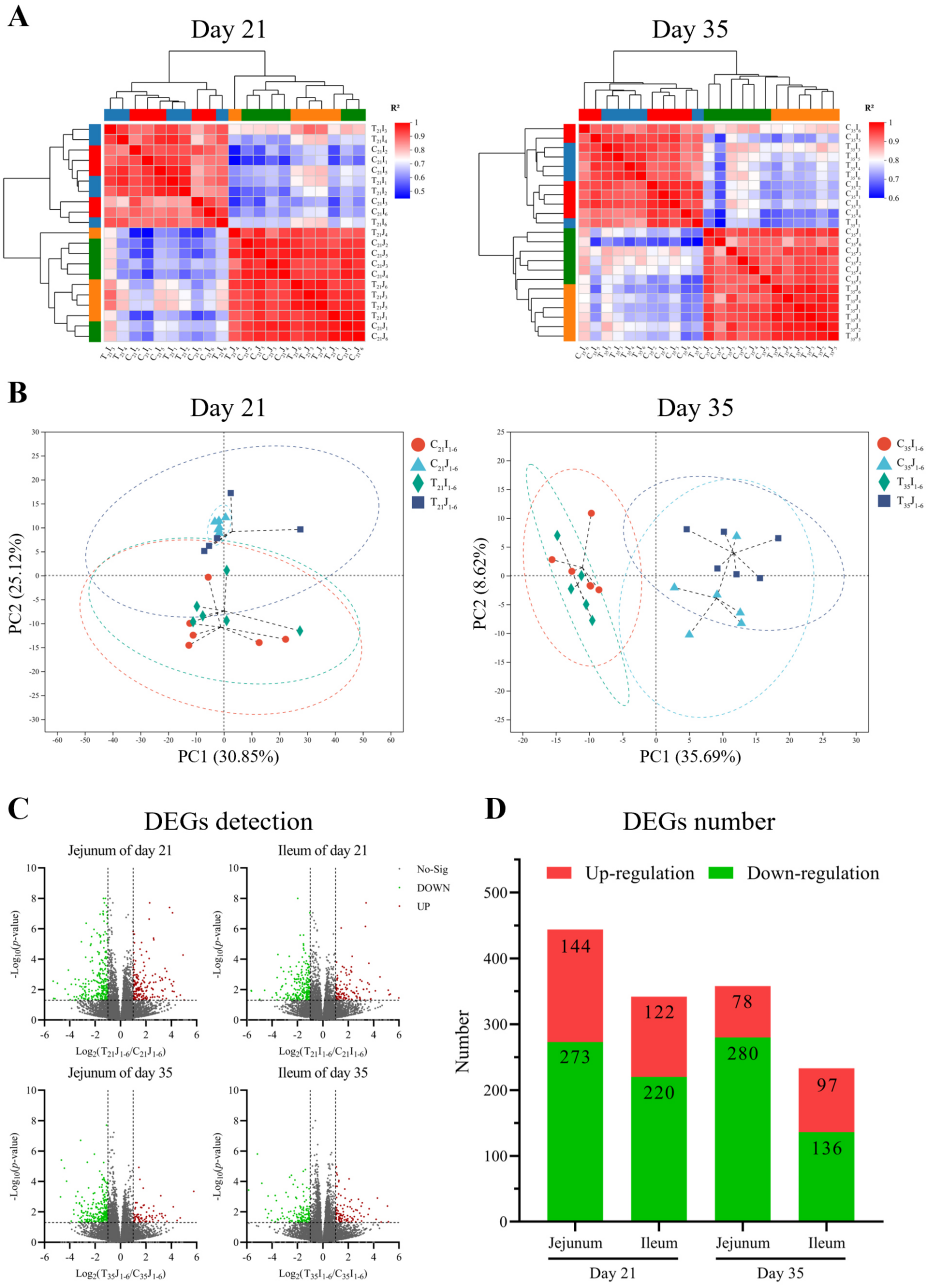
Inter-sample analyses of chicken jejunum and ileum at day 21 and day 35, respectively. **(A)** Heatmap of Pearson correlation analysis. **(B)** Principal component analysis (PCA) cluster correlation analysis. **(C)** Statistical volcano plot of differentially expressed genes (DEGs). **(D)** Number of DEGs.

Correlation analysis was conducted on the transcriptome data. Pearson correlation analysis between samples using average hierarchical clustering and Euclidean distance algorithm computation indicated that the Pearson coefficients for jejunum and ileum tissue at 21 d exceeded 0.87 and 0.85, respectively, and over 0.87 and 0.88 at 35 d, respectively (**Figure 2A**). The results of principal component analysis (PCA) revealed that the major coverage for jejunum and ileum tissue was 55.97% at 21 d and 44.31% at 35 d, respectively (**Figure 2B**).

Differential expressed genes (DEGs) analysis of sequencing data illustrated that in comparison to control group, B.A.-TL group at 21 d of age exhibited a total of 171 upregulated genes and 273 downregulated genes in the jejunum samples, while the ileum samples showed 122 upregulated genes and 220 downregulated genes (**Figures 2C, D**). At 35 d of age, the jejunum samples of B.A.-TL group displayed 78 upregulated genes and 280 downregulated genes, and the ileum samples showed 97 upregulated genes and 136 downregulated genes. Specifically, the key DEGs associated with immune regulation at 21 d of age included *CCL4*, *CCL26*, *IL-1β*, *IL-26*, *IL-8L1 (IL-8)*, *LITAF*, *IFNG*, *IRF (1, 5, 7, 9)*, *MX1*, *TNFSF (8, 10, 13B, 15)*, *LYZ*, *CCR5*, *TLR3*, and *TLR4*. At 35 d of age, DEGs related to immune regulation mainly contained *IL-6*, *IL-8*, *LYZ*, and *XCL1*.

**Figure 3:**
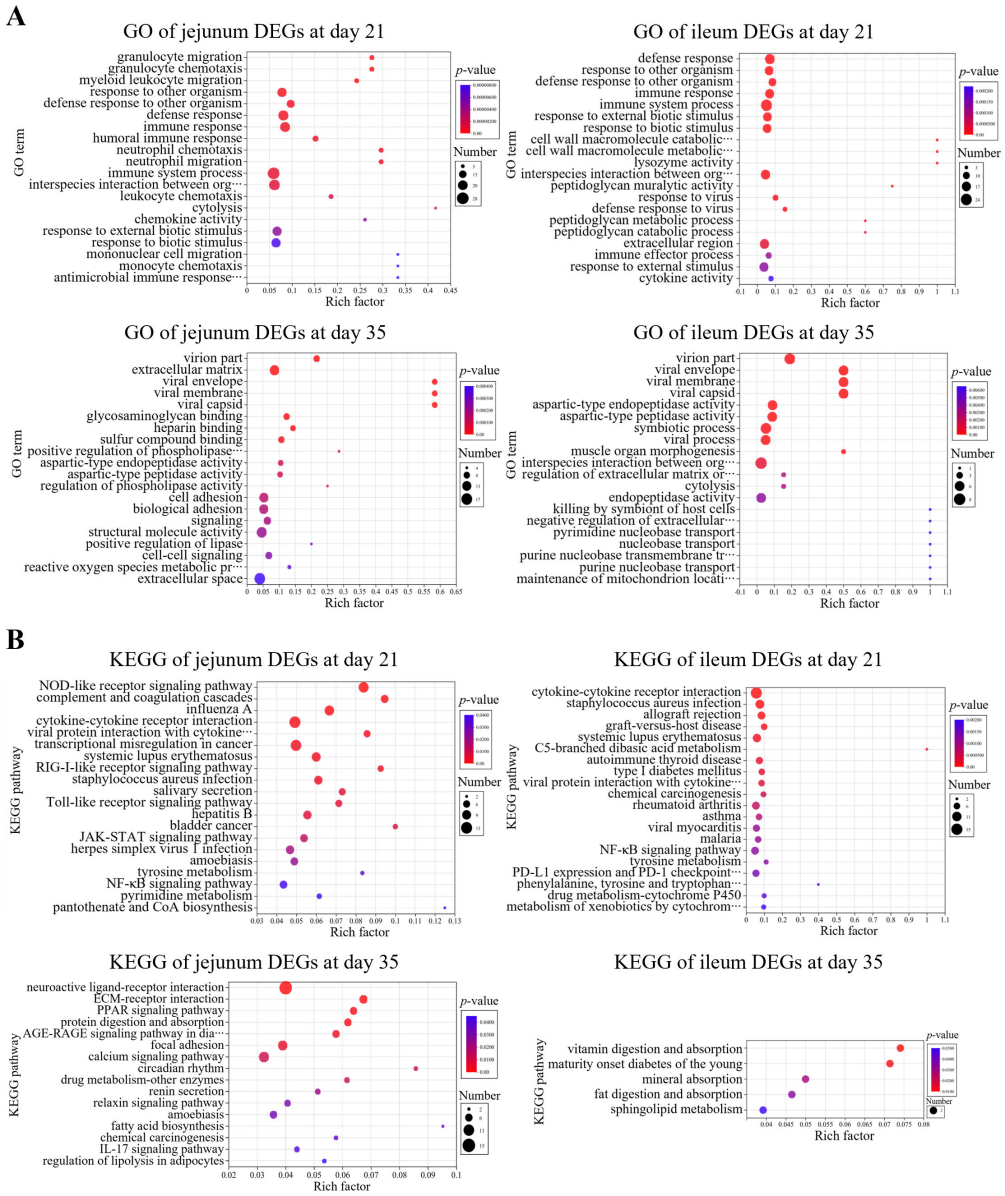
Enrichment analysis of differentially expressed genes (DEGs). **(A)** GO and **(B)** KEGG enrichment analyses of DEGs of jejunum and ileum samples at day 21 and day 35, respectively.

GO enrichment analysis of DEGs was performed using the Benjamini-Hochberg multiple testing correction method (*p* < 0.05) (**Figure 3A**). The results showed that in comparison to control group, the 21-day-old broiler DEGs in jejunum samples were mainly related to GO terms of cell chemotaxis, migration, and immune response (downregulation); the DEGs in ileum samples were primarily related to immune response (downregulation). The 35-day-old broiler DEGs in jejunum samples were related to the GO terms in the cellular component category (downregulation); while the DEGs in ileum samples were enriched into GO terms in many biological processes.

KEGG enrichment analysis of DEGs was performed using the Benjamini-Hochberg multiple testing correction method (*p* < 0.05) (**Figure 3B**). The results showed that in comparison to control group, the 21-day-old broiler DEGs in jejunum samples exhibited enrichment in 21 KEGG signaling pathways (downregulation), e.g., cytokine-cytokine receptor interaction, chemokine signaling pathway, and NF-κB signaling pathway; the DEGs in ileum samples showed enrichment in 48 KEGG signaling pathways (downregulation), e.g., cytokine-cytokine receptor interaction, NF-κB signaling pathway, chemokine signaling pathway, Th1 and Th2 cell differentiation, natural killer cell-mediated cytotoxicity, T cell receptor signaling pathway, and Toll-like receptor signaling pathway. However, the 35-day-old broiler DEGs in jejunum or ileum samples were not significantly enriched in immune- or inflammation-related pathways.

The validation of RNA-sequencing was provided in **Supplementary Figure 2** by qRT-PCR with *GAPDH* as the internal reference gene.

### 3.4 Analysis of secondary metabolites of *Bacillus amyloliquefaciens* TL

**Figure 4:**
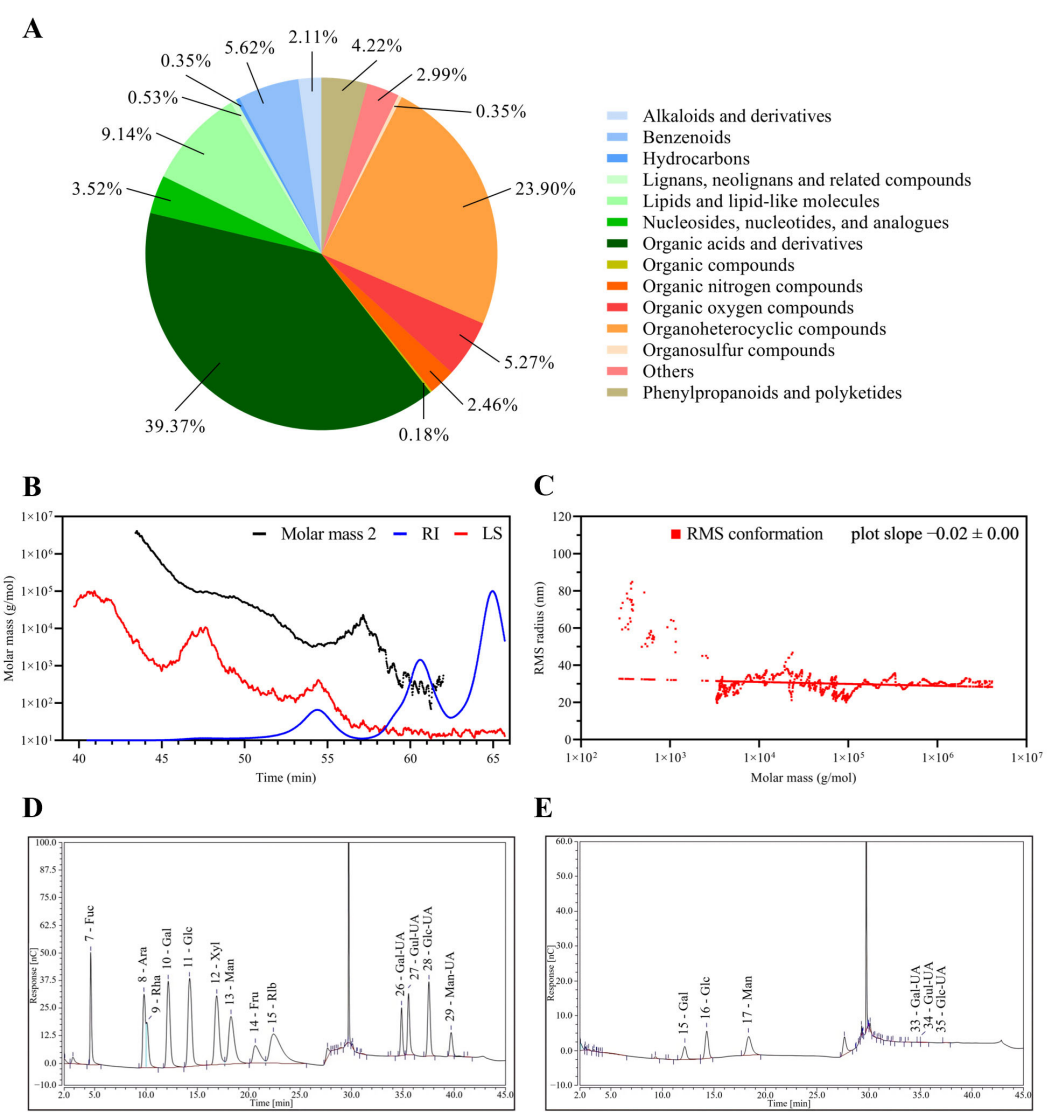
Composition analysis of the secondary metabolites from *Bacillus amyloliquefaciens* TL fermentation supernatant (FMTs). **(A)** Classification and statistics of metabolites from *B*. *amyloliquefaciens* TL. **(B)** Absolute molecular weight analysis of extracellular polysaccharides from *B*. *amyloliquefaciens* TL (EPS-TL). **(C)** Molecular configuration analysis of EPS-TL from *B*. *amyloliquefaciens* TL. **(D)** Monosaccharide standard chromatogram. **(E)** Monosaccharide composition of EPS-TL.

The metabolomic profiling of the fermentation supernatants of B.A.-TL revealed predominant categories of metabolites (**Figure 4A**). The relative molecular weight of EPS-TL was determined (**Figure 4B**). EPS-TL exhibited a weight-average molecular weight (Mw) of 5.16 kDa, and a polydispersity index of 1.09 (**Figure 4C**). The results of monosaccharide composition revealed that EPS-TL was mainly composed of three monosaccharides, i.e., galactose (Gal, 18.28%), glucose (Glc, 29.02%), and mannose (Man, 50.26%), with a molar ratio of Gal: Glc: Man = 1:4.3:7.5 (**Figures 4D, E**); 1 L of probiotic fermentation liquid yielded approximately 1.24 mg of EPS-TL. EPro-TL crude product was obtained with the concentration of approximately 1303.20 μg/mL. A yield of 21.72 mg of EPro-TL was obtained from 1 L of probiotic fermentation liquid.

### 3.5 Effects of *Bacillus amyloliquefaciens* TL cells, supernatant, extracellular polysaccharides, and extracellular proteins against LPS on HD11 cell proliferation

**Figure 5:**
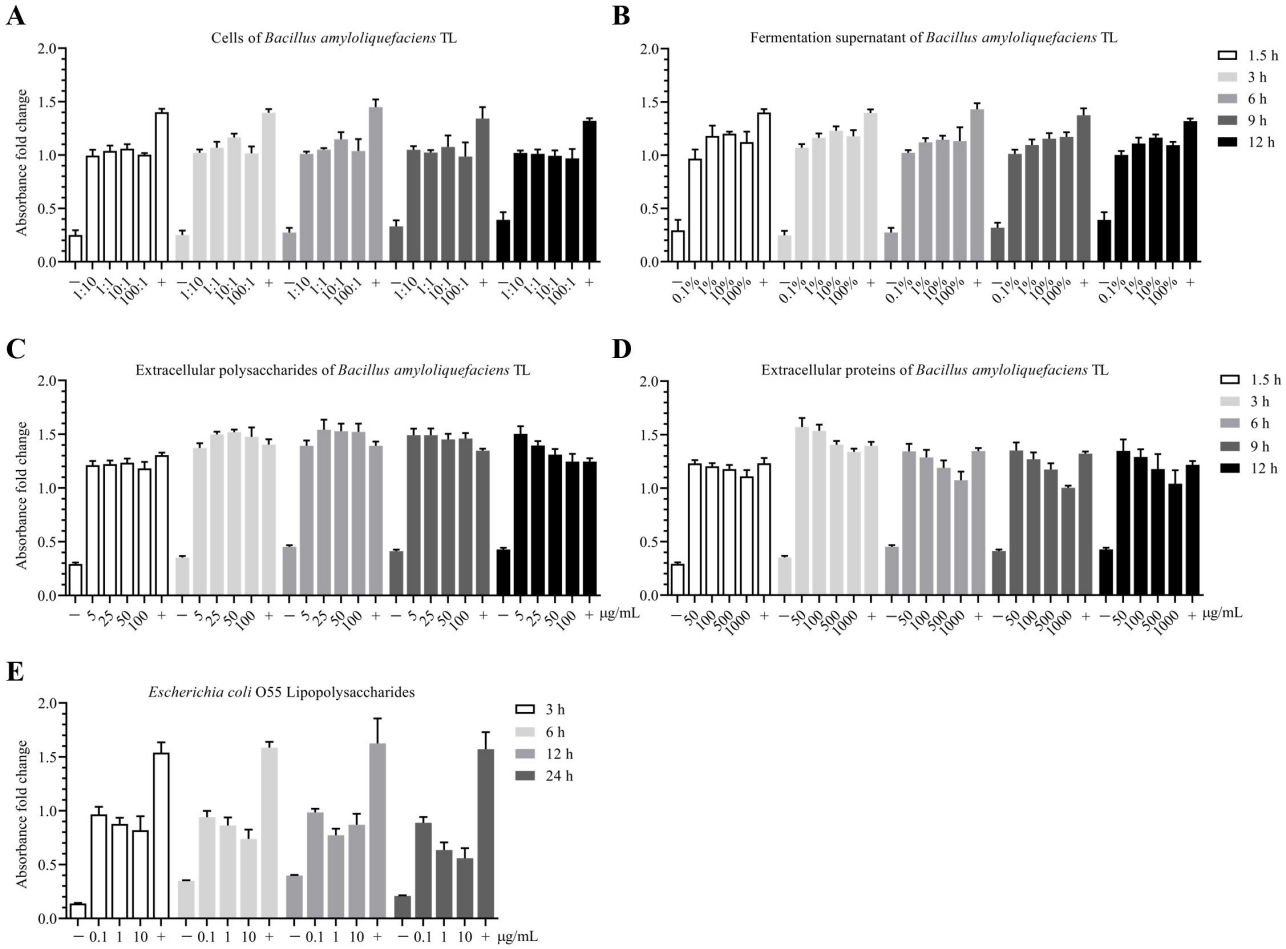
CCK-8 toxicity assay of *Bacillus amyloliquefaciens* TL and effects of its secondary metabolites on HD11 cell proliferation. **(A)** Concentration-time gradient effect of *B*. *amyloliquefaciens* TL cells on the proliferation of HD11 cells based on cell number ratio (n:n), with bacterial cells having an initial concentration at OD_450_ = 1.0 (approximately 1.5 × 10^8^ CFU/mL). **(B)** Concentration-time gradient effect of *B*. *amyloliquefaciens* TL fermentation supernatant (FMTs) on HD11 cell proliferation. FMTs with an initial concentration at OD_450_ = 1.0. **(C)** Concentration-time gradient effect of *B*. *amyloliquefaciens* TL extracellular polysaccharides (EPS-TL) on HD11 cell proliferation. **(D)** Concentration-time gradient effect of *B*. *amyloliquefaciens* TL extracellular proteins (EPro-TL) on HD11 cell proliferation. **(E)** Concentration-time gradient effect of *Escherichia coli* O55 lipopolysaccharides (LPS) on HD11 cell proliferation. “−” and “+” indicate negative (DMEM) and positive (DMEM with 10% fetal bovine serum) controls, respectively. 6 replicates in each data.

The proliferation toxicity of B.A.-TL components and LPS on HD11 cells (approximately 1.5 × 10^6^ cells/well in 6-well plate with 80% cell confluence) were performed. When the B.A.-TL to HD11 cells ratio (n/n) reached approximately 100:1, and 1 mL of 1.0 OD_450_ bacterial suspension (no dilution, approximately 1.50 × 10^8^ CFU/mL) was used for bacterial treatment, a slight inhibitory effect on cell growth was observed after 12 h (*p* > 0.05). However, as the ratios were increased to 10:1 (1/10 dilution), 1:1 (1/100 dilution), and 1:10 (1/1000 dilution), no inhibitory effects were observed (**Figure 5A**). The supernatant corresponding to the bacterial concentration did not exhibit cytotoxic effects across the HD11 concentration-time gradients (**Figure 5B**); EPS-TL promoted HD11 cell growth at different concentrations and stimulation times (**Figure 5C**), and EPro-TL exhibited similar stimulatory effects on HD11 cells as EPS-TL. However, with increasing EPro-TL concentration and stimulation time, the promotion of HD11 cell growth showed a gradual decline (**Figure 5D**). Different LPS stimulation concentrations and times inhibited the growth of HD11 cells (**Figure 5E**), with the most significant inhibitory effect observed at 10 μg/mL LPS stimulation.

### 3.6 Alleviation of LPS-induced high inflammatory factor expression in HD11 cells by *Bacillus amyloliquefaciens* TL cells, supernatant, extracellular polysaccharide, and extracellular proteins

**Figure 6:**
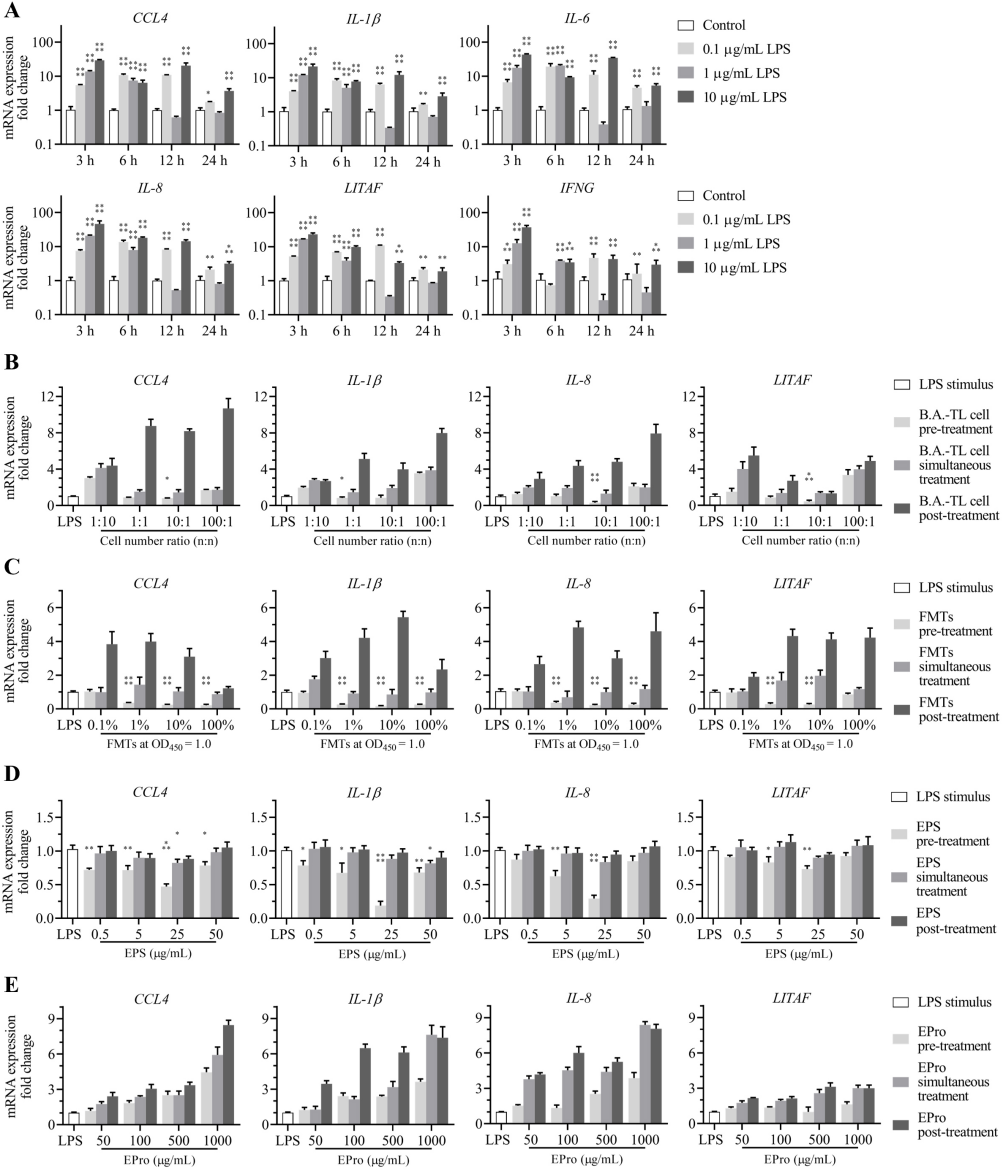
Alleviation effects of *Bacillus amyloliquefaciens* TL and its secondary metabolites on the elevated mRNA expression of inflammatory factors in HD11 cells induced by *Escherichia coli* O55 lipopolysaccharides (LPS) stimulus. **(A)** mRNA expressions of inflammatory factors from HD11 cells induced by LPS concentration-time gradient stimulus. **(B)** *B*. *amyloliquefaciens* TL cells (with an initial concentration at OD450 = 1.0, approximately 1.5 × 10^8^ CFU/mL), **(C)** fermentation supernatants (FMTs), **(D)** extracellular polysaccharides (EPS-TL), and **(E)** extracellular proteins (EPro-TL) are treated against LPS at different concentrations through pre-treatment, simultaneous treatment, and post-treatment, respectively, on HD11 cells to alter the mRNA expression of inflammatory factors. 6 replicates in each data. *p*<0.05(*), *p*<0.01(**), *p*<0.001(***), and *p*<0.0001(****).

The LPS was used to stimulate the HD11 cells at various concentrations and treatment times. Based on the RNA-Seq analysis, the expression of inflammatory factors in cells was detected. The results revealed that stimulation of HD11 cells with 10 μg/mL LPS for 3 h led to a 10^1^–10^2^-fold upregulation in the mRNA expression of inflammatory factors *CCL4*, *IL-1β*, *IL-6*, *IL-8*, *LITAF*, and *IFNG*, establishing a cellular model with a heightened inflammatory response (**Figure 6A**).

HD11 cells were pre-treated with B.A.-TL cells and supernatant, followed by LPS stimulation, and the expression of inflammatory factors was measured. The results showed that pre-treating HD11 cells with B.A.-TL cells (TL: HD11 cells = 10:1 or 1:1; n/n) and supernatant (1/10 and 1/100 dilution) effectively inhibited the elevation of inflammatory factor expression (**Figure 6B**). However, simultaneous treatment of B.A.-TL cells or supernatant with LPS or post-treatment of HD11 cells after LPS stimulation did not suppress the enhanced levels of inflammatory factor expression.

HD11 cells were respectively treated with EPS-TL or EPro-TL in combination with LPS, and the expression of inflammatory factors was measured. The results showed that pre-treating HD11 cells with different concentrations of EPS-TL effectively inhibited the elevation of inflammatory factor (*CCL4*, *IL-1β*, *IL-8*, and *LITAF*) expression induced by LPS (*p* < 0.05) (**Figure 6C**). Simultaneous treatment or post-treatment of HD11 cells with EPS-TL and LPS stimulation was less protective than pre-treatment with EPS-TL and LPS stimulation. Either pre-treatment, simultaneous treatment, or post-treatment of EPro-TL with different concentrations did not effectively inhibit the high expression of inflammatory factors induced by LPS, and the mRNA expression of inflammatory factors *CCL4*, *IL-1β*, *IL-8*, and *LITAF* exhibited varying degrees of upregulation.

### 3.7 ELISA detection of inflammatory factors in cells

**Figure 7:**
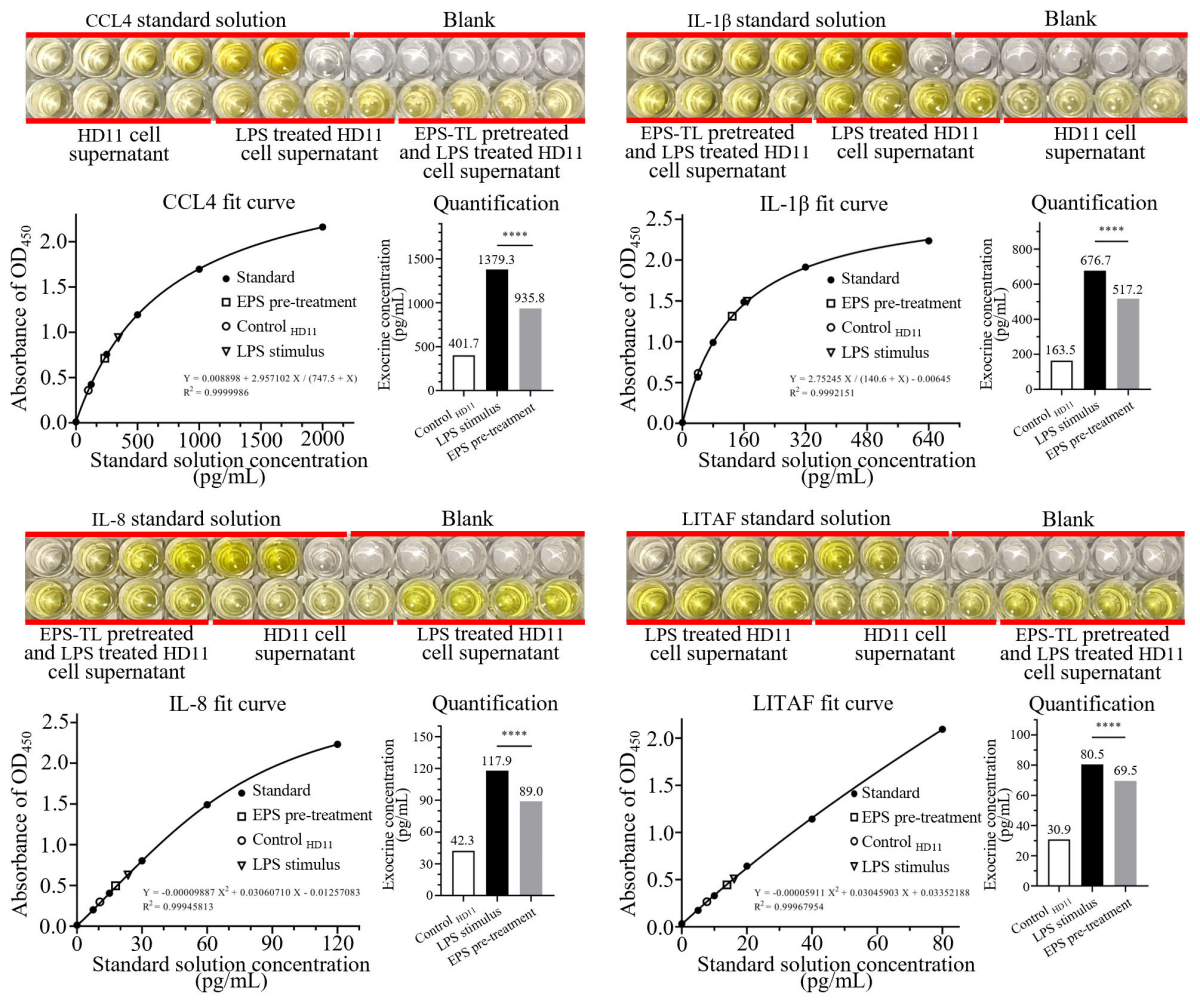
ELISA detection of inflammatory factors CCL4, IL-1β, IL-8, and LITAF in HD11 cell supernatant under different conditions. 4 replicates in each data. *p*<0.0001(****).

HD11 cells were treated with EPS-TL and LPS as described in Section 2.8, and the changes of inflammatory factors in the cell supernatant were quantitatively measured. A fitted curve was constructed based on the OD_450_ absorbance. The results demonstrated that compared to direct LPS stimulation, pre-treating HD11 cells with EPS-TL significantly reduced the elevation of inflammatory factors, including CCL4, IL-1β, IL-8, and LITAF (**Figure 7**). However, the levels of inflammatory factors in the cell supernatant from both the EPS-TL pre-treatment group and the LPS treatment group were still significantly higher than those in normal HD11 cells.

**Figure 8:**
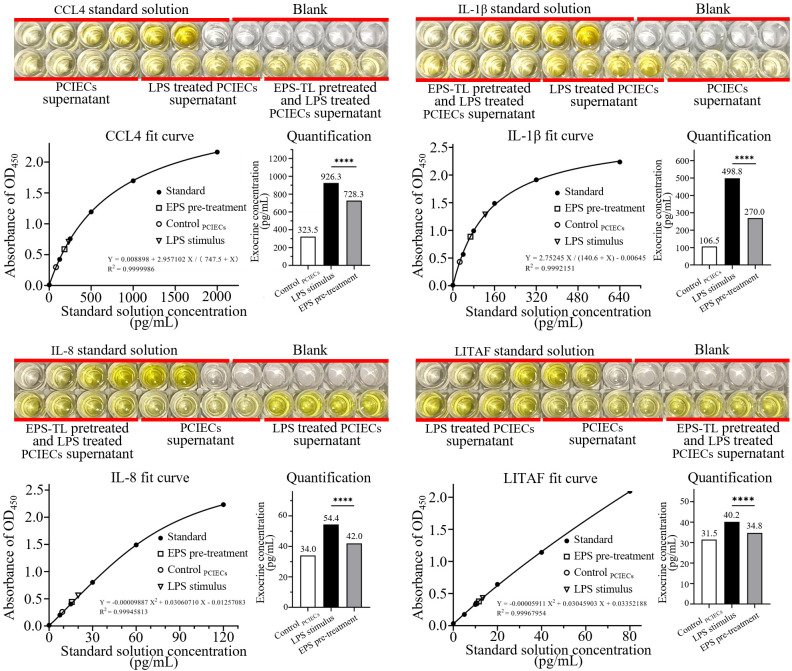
ELISA detection of inflammatory factors CCL4, IL-1β, IL-8, and LITAF in the supernatant of primary chicken embryonic small intestinal epithelial cells (PCIECs) under different conditions. 4 replicates in each data. *p*<0.0001(****).

The PCIECs (morphology and identification were provided in **Supplementary Figure 3**) were treated with EPS-TL and LPS as described in Section 2.8. The expression of inflammatory factors in the primary cells were detected. The LPS stimulation still elevated the mRNA expression of inflammatory factors, and the content of inflammatory factors in the supernatant of primary cells was increased by 2–4 times (**Figure 8**). However, pre-treatment of primary cells with EPS-TL effectively inhibited the increase in the expression of inflammatory factors CCL4, IL-1β, IL-8 and LITAF, which was consistent with the results of the HD11 cell experiments. Moreover, compared to LPS, pre-treatment of primary cells with EPS-TL reduced the content of inflammatory factors in the supernatant by more than 10%.

### 3.8 Localization and phosphorylation analysis of main factors in NF-κB signaling pathway of HD11 cells after treatment with EPS-TL and LPS

**Figure 9:**
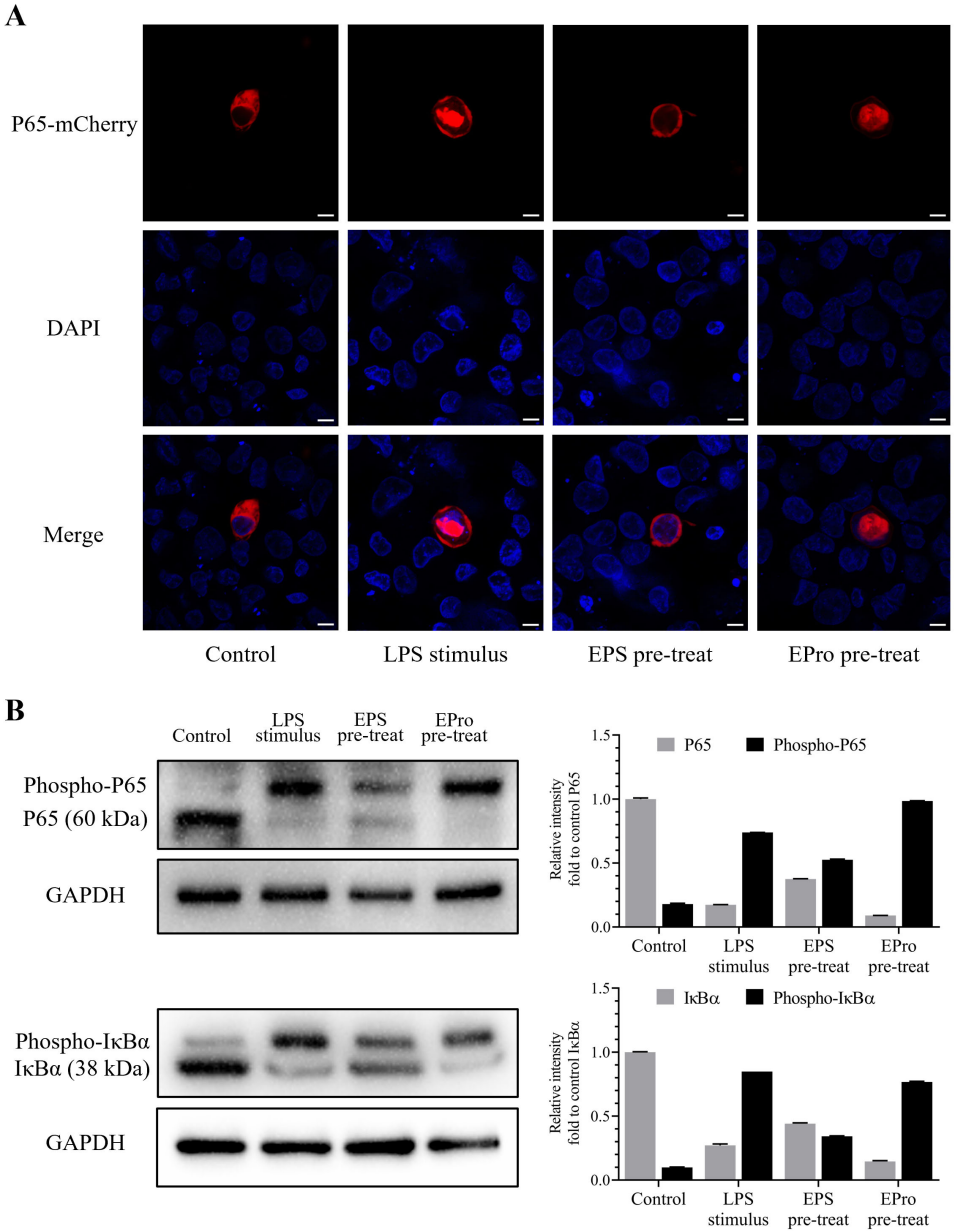
Localization and phosphorylation detection of the terminal proteins IκBα and P65 evaluating the activation of NF-κB signaling pathway in HD11 cells. **(A)** Localization of NF-κB P65 nuclear factor coupled with mCherry fluorescence in HD11 cells under different treatments. **(B)** Visualization and quantification of normal and phosphorylated IκBα and P65 proteins in HD11 cells under different treatments. The scales in figures are 10 nm.

HD11 cells were treated with EPS-TL and LPS as described in Section 2.8, and the cellular fluorescence was observed. The results showed that compared to normal HD11 cells, after treatment with LPS, nuclear factor P65 was significantly translocated into the nucleus, with fluorescence mainly concentrated in the nucleus, and only a small amount of fluorescence in the cytoplasm (**Figure 9A**). In contrast, pre-treatment of HD11 cells with EPS-TL showed that LPS could induce partial translocation of nuclear factor P65 into the nucleus compared to normal HD11 cells, but there was still a portion of the nuclear factor distributed around the nucleus without translocation into the nucleus. These observations suggested that pre-treatment with EPS-TL could mitigate the translocation of nuclear factor P65 induced by LPS.

After implementing Phos-tag™ Acrylamide AAL-107 reagent to prepare concentrated and separated gels, the migration rate of phosphorylated IκBα and P65 protein in the gel were evidently slowed down, distinguishing the phosphorylated protein band and ordinary protein band and facilitating the identification of protein phosphorylation. The results indicated that under untreated conditions, HD11 cells only exhibited a slight amount of NF-κB signaling pathway activation, with the unphosphorylated IκBα and P65 protein content significantly higher than that of phosphorylated protein (*p* < 0.001) (**Figure 9B**). LPS stimulation significantly induced activation of the classical NF-κB signaling pathway (*p* < 0.001), leading to a significant conversion in the content of both phosphorylated and unphosphorylated IκBα and P65 proteins. After pre-treatment with EPS-TL, HD11 cells showed a reduction in the proportion of phosphorylation of both types of proteins along with the decrease in the level of IκBα and P65 protein phosphorylation.

## 4 Discussion

### 4.1 Functional analysis of *Bacillus amyloliquefaciens* TL in broiler growth and intestinal development

The taxonomic composition and relative abundance of intestinal microbiota play crucial roles in the growth and development of animals. Probiotic supplementation can aid in the establishment of a healthy intestinal microbiota, thereby promoting positive structural and functional changes. Early supplementation of probiotics during animal growth and development is particularly beneficial, as it can help prevent the colonization of harmful microorganisms in the digestive tract.

This study demonstrated that B.A.-TL supplementation significantly enhanced the growth performance of Cobb500 broilers. However, these benefits were mostly pronounced in the early stages of broiler development (optimum between 14–21 d of age), with a gradual decline in efficacy thereafter. This observation is consistent with previous studies on probiotics such as *B. amyloliquefaciens* KB54 (4) and *B. subtilis* ATCC19659 (46), indicating a common pattern in promoting broiler weight accumulation. The observed phenomenon could be attributed to two primary factors. First, as animals mature, the intestinal microbiota tends to establish a relatively stable community structure through interactions among microbial communities and reciprocal selection with the host organism. Significantly disrupting this dynamic equilibrium structure is generally difficult for B.A.-TL. Second, as the intestinal microbiota matures and the bacterial population increases, the presence of B.A.-TL cells in the intestine may have a decreased impact on the existing microbial abundance, leading to a decline in the observed beneficial effects over time.

The growth of intestinal villi, as indicated by Vh and VCR, is a crucial reflection for gastrointestinal health, indicating the surface area available for nutrient absorption in the gastrointestinal tract (49). In this study, B.A.-TL supplementation significantly enhanced the growth of intestinal villi in broiler small intestine tissue, suggesting a positive influence on the digestion and absorption of nutrients.

### 4.2 Functional analysis of *Bacillus amyloliquefaciens* TL in gene expression of broiler intestine

The Pearson correlation analysis between samples revealed strong biological reproducibility of both B.A.-TL and control groups. The PCA results highlighted notable patterns in gene expression within and between the jejunum and ileum samples at different ages. As animals mature, the differences in gene expression patterns within the same intestinal segment gradually decrease, while different intestinal segments increase, suggesting that the influence of B.A.-TL on gene expression patterns in the small intestine gradually diminishes, and as the organs mature, differences in gene expression patterns between the jejunum and ileum become more pronounced.

The results of DEGs analysis revealed significant downregulation of genes related to intestinal immunity and inflammatory responses, including CCL4, IL-1β, IL-8, and LITAF, which play crucial roles in regulating innate and adaptive immune responses, as well as mediating inflammatory reactions in poultry. The inflammatory reactions are invariably accompanied by the heightened expression and recruitment of inflammatory factors. CCL4 is involved in nonspecific immunity (13) by clearing bacterial endotoxins and viral particles (50). IL-1β is a multifunctional inflammatory factor, which is essential for regulating the occurrence and development of viral diseases in poultry (51–53). IL-8 serves as an efficient chemotactic agent for heterophils and macrophages (54), which plays an indispensable role in poultry diseases and inflammation (55, 56). LITAF, recognized as a chicken TNF-α substitute, is expressed in various avian leukocytes and lymphocyte subsets (15). The downregulation of these cytokines suggests an evident modulation of the inflammatory response while in the healthy status.

Both GO and KEGG enrichment analyses of DEGs revealed significant enrichment of inflammation-related biological functions, which suggest that B.A.-TL reduces intestinal tissue inflammation to harmful substances present in poultry intestine, including defense against bacteria, viruses, and inflammatory processes, particularly at 21 d of age. However, the downregulation of inflammatory factors could still be observed at 35 d of age, while related biological processes could no longer be enriched. The results indicate that B.A.-TL is more effective in coordinating the intestinal microbiota with the host’s body in the early stages of broiler development, enhancing the tolerance of broiler intestinal contents. The KEGG enrichment revealed that the cytokine-cytokine receptor interaction signaling pathway is significantly enriched, encompassing a wide range of cytokines and receptors involved in signal transduction processes in intestinal tissues, which could be frequently enriched in immune regulation and inflammatory responses (57, 58). Additionally, the NF-κB signaling pathway, which presents in almost all somatic cells, is significantly enriched. This pathway is involved in the cellular response to various stimuli, including inflammatory activation, in addition to bacteria, viruses, parasites, heat stress, ultraviolet radiation, and chemicals (59–61). Probiotic B.A.-TL help mitigate inflammatory responses and maintain intestinal function, ultimately resulting in a low inflammatory state and improved intestinal health and benefiting broiler growth and performance.

### 4.3 Functional analysis of *Bacillus amyloliquefaciens* TL cells and its secondary metabolites in preventing cellular inflammation

This study delved into understanding the inhibition of B.A.-TL in inflammatory responses by examinations on HD11 cells. The results demonstrated that neither the bacterial cells, supernatant, nor the secondary metabolites, i.e., EPS-TL and EPro-TL, displayed cytotoxic effects on HD11 cells within the specified concentration-time gradient, which suggest that B.A.-TL possesses characteristics that promote cell proliferation without inducing cytotoxicity.

This study uncovered findings regarding the potentially anti-inflammatory components of B.A.-TL, distinguishing between bacterial cells and supernatant on HD11 cells when subjected to LPS stimulation. It was observed that pre-treating HD11 cells with B.A.-TL supernatant effectively inhibited the gene upregulation of inflammatory factors induced by LPS. However, simultaneous treatment or post-treatment with supernatant or bacterial cells resulted in significantly higher levels of inflammatory factors compared to the LPS treatment group, demanding further exploration of the underlying mechanisms.

The subsequent research focused on EPS-TL and EPro-TL, which was isolated from the mixed system of probiotic supernatants, respectively, for better understanding of the anti-inflammatory activity produced by B.A.-TL. The results revealed that EPS-TL exhibited significant anti-inflammatory characteristic. Furthermore, Western blot assay suggested an alleviated activation of NF-κB signaling pathway and quantitative ELISA detection confirmed the reduction in inflammatory factors, which was consistent with changes in gene expressions. Notably, EPS-TL also demonstrated anti-inflammatory properties in PCIECs. However, EPro-TL did not show any anti-inflammatory potential in this study.

## 5 Conclusion

This study highlights the promotion of dietary intake B.A.-TL on the growth performance and intestinal health of Cobb500 broilers. B.A.-TL exerts a profound influence on gene expression within the small intestine. And one of the extracellular polysaccharide components of B.A.-TL, EPS-TL, was identified as a primary contributor to its anti-inflammatory properties, which demonstrates the ability to significantly mitigate the elevated expression of inflammatory factors induced by LPS in both HD11 cells and PCIECs. These fundings reveal EPS-TL as a biologically active molecule with promising anti-inflammatory potential among the secondary metabolites produced by probiotic B.A.-TL.

## Data Availability

The datasets presented in this study can be found in online repositories. The names of the repository/repositories and accession number(s) can be found below: https://www.ncbi.nlm.nih.gov/, PRJNA1102261.
