# Supplementary material for: Bacillus amyloliquefaciens TL promotes gut health of broilers by the contribution of bacterial extracellular polysaccharides through its anti-inflammatory potential
Source: Front Immunol. 2024 Sep 23;15:1455996. doi: 10.3389/fimmu.2024.1455996 (PMC11456473; doi:10.3389/fimmu.2024.1455996)
Supplement: Supplementary file 1 [file DataSheet1.docx]

Supplementary Material

# Materials and methods

## Analysis of fermentation supernatant from *Bacillus amyloliquefaciens* TL

The fermentation supernatant of B.A.-TL underwent collection and sterilization through filtration. Target compounds were separated utilizing a Vanquish ultra-high-performance liquid chromatography (UHPLC; Thermo Fisher Biotechnology Co., Ltd., MA, USA) system equipped with a Waters ACQUITY UPLC BEH Amide column (1.7 µm of particle size with 2.1 mm × 100 mm of volume and 1/pk of units per package). Mass spectrometry (QE HFX) was employed in Information-Dependent Acquisition (IDA) mode to gather MS/MS spectra. Raw data in mzXML format were processed to obtain peak identification, extraction, alignment, and integration, employing a custom R package (XCMS kernel). Substance annotation was performed against the self-constructed BiotreeDB (V2.1) secondary mass spectrometry database, with a cutoff score of 0.3.

## Raising condition of experimental animals

The animals were given 24 h to acclimate to the housing environment. The experiment was carried out in cages, and chicks had ad libitum access to feed and water throughout the study, with feed supplied three times a day. The housing facility was equipped with fans for ventilation and heating. The temperature was maintained at 33–35 °C from 0 to 7 d, followed by a gradual decrease of 2 °C per week. The lighting schedule entailed 24 h of light in 1–3 d, 22 h in 4–7 d, 18 h in 8–21 d, and 16 h in 22–35 d. Humidity in the facility was controlled at 70% from 0 to 7 d and 55–65% from 8 to 35 d.

## Tissue sampling and sectioning

At 21- and 35-day-old, chickens with individual body weights close to the average of each group were chosen and euthanized using cervical dislocation. The duodenum, jejunum, and ileum tissues were collected, meticulously cleaned, with half of each tissue sample stored in tissue cryotubes and the other half fixed in 4% paraformaldehyde (BioSharp Biotechnology Co., Ltd., Hefei, China). Cecal contents were directly collected into sterile cryotubes and immediately frozen in liquid nitrogen for storage.

The fresh intestine tissues were fixed in 4% paraformaldehyde for at least 24 h, and the target tissues were evenly trimmed using a surgical knife within a fume hood. The trimmed tissues were labeled and arranged in a dehydrating box, which was then placed in a basket and sequentially dehydrated in a dehydrator. Following dehydration, the tissues underwent sectioning and H&E staining. The slides were sealed with neutral resin for preservation.

## RNA extraction, cDNA library preparation and quantitative real-time PCR

Total RNA extraction from cells and tissue samples was conducted using the Total DNA/RNA Isolation Kit (Omega agent Biotechnology Co., Ltd., Guangzhou, China), and the synthesis of the first-strand cDNA was performed using the HiScript III 1st Strand cDNA Synthesis Kit (Vazyme Biotechnology Co., Ltd., Nanjing, China) following the manufacturers’ instructions.

Primers used in the qRT-PCR were provided in Supplementary Table 2. The cDNA was diluted 10 times, and SYBR qPCR MIX fluorescent quantitative reagent (Vazyme Biotechnology Co., Ltd., Nanjing, China) was utilized for qRT-PCR analysis on the CFX Connect^TM^ system (Bio-Rad Biotechnology Co., Ltd., CA, USA). The expression of target genes was assessed using the *GAPDH* gene as an internal reference, and each experiment was repeated with three biological replicates. The PCR amplification program included an initial denaturation at 95 °C for 3 min, followed by 40 cycles of denaturation at 95 °C for 10 sec and annealing at 60 °C for 30 sec. Subsequently, the temperature was gradually increased to 95 °C, then decreased to 60 °C, and finally raised to 95 °C, with fluorescence signal captured every 0.05 sec and a melt curve generated.

## Illumina sequencing, quality control and analysis

A total of 48 tissue samples , i.e., six biological replicates each of the ileum and cecum tissues from both B.A.-TL and control group at 21 and 35 d of age, respectively, underwent preliminary quality control analysis before sequencing. Total RNA was extracted from all samples, and the RNA concentration and purity were assessed using the NanoDrop 2000c spectrophotometer (Thermo Fisher Biotechnology Co., Ltd., MA, USA), with OD260/OD280 ratios ranging from 1.9 to 2.1 indicating acceptable quality. Qualified samples underwent sequencing on the Illumina second-generation high-throughput sequencing platform (Majorbio Biotechnology Co., Ltd., Shanghai, China). The raw RNA sequencing data were subjected to quality control using both SeqPrep (<https://github.com/jstjohn/SeqPrep/>; accessed on 10 Junuary 2022) and Sickle (<https://github.com/najoshi/sickle/>; accessed on 10 Junuary 2022). Following quality control, the clean reads were aligned to the reference genome of chicken (*Gallus gallus*; GCF_016700215.1) to obtain mapped reads for subsequent transcriptome assembly and expression level calculation. The quality of this transcriptome sequencing, including sequencing saturation, gene coverage, distribution of reads in different regions of the reference genome, and reads distribution across different chromosomes, was evaluated using TopHat2 (https://ccb.jhu.edu/software/tophat/index.shtml; accessed on 20 Junuary 2022).

The stability of gene expression levels in each sample was evaluated using MAPlot software (1). To ensure comparability of gene expression levels estimated between different genes and experiments, transcripts per million reads (TPM) were utilized for a more intuitive comparison of gene expression levels. Differentially expressed genes (DEGs) were identified with filtering criterion of *p* < 0.05 and |Log_2_(Fold Change)| ≥ 1. The significant enrichment of DEGs based on Gene Ontology (GO; <http://www.geneontology.org/>; accessed on 10 Feburary 2022) or Kyoto Encyclopedia of Genes and Genomes (KEGG; <https://www.genome.jp/kegg/>; accessed on 10 Feburary 2022) was determined by cluster analysis and profile exact testing (*p* < 0.05).

**References**

1. Wang, L., Feng, Z., Wang, X., Wang, X., & Zhang, X. (2010). DEGseq: an R package for identifying differentially expressed genes from RNA-seq data. Bioinformatics, 26, 136–138. doi: 10.1093/bioinformatics/btp612

# Supplementary Figures and Tables

**Supplementary Table 1** Basal diet composition

| **Ingredient and nutrient** | **Mixture ratio and content** |
| --- | --- |
| 1–21 d | |
| Corn, yellow | 59% |
| Soybean meal, dehulled | 31% |
| Fish meal | 2.5% |
| Wheat bran | 1.45% |
| Salt | 0.3% |
| Soybean oil | 2.5% |
| DL-methionine | 0.15% |
| Limestone | 1.1% |
| Calcium hydrogen phosphate | 1.4% |
| Mineral and vitamine premixa^a^ | 0.6% |
| Total | 100% |
| ME | 12.4 MJ/kg |
| Crude protein | 19.8 g/kg |
| Calcium | 0.896 g/kg |
| Phosphorus | 0.6 g/kg |
| Lysine | 1.16 g/kg |
| Methionine | 0.36 g/kg |
| Methionine and cysteine | 0.63 g/kg |
| 21–35 d | |
| Corn, yellow | 62.4% |
| Soybean meal, dehulled | 27.23% |
| Soybean oil | 4.5% |
| Phosphorus | 1.2% |
| Fish meal | 2.5% |
| Salt | 0.2% |
| Limestone | 1.0% |
| Mineral and vitamine premixa^a^ | 0.58% |
| DL-methionine | 0.24% |
| Lysine | 0.15% |
| Total | 100% |
| ME | 12.97 MJ/kg |
| Crude protein | 19.4 g/kg |
| Calcium | 1.05 g/kg |
| Phosphorus | 0.59 g/kg |
| Lysine | 1.64 g/kg |
| Methionine | 0.53 g/kg |
| Methionine and cysteine | 0.85 g/kg |

Note: “^a^:” supplied per kilogram diet. Zinc: 80 mg; iron: 80 mg; copper: 8 mg; iodine: 0.45 mg; selenium: 0.20 mg; manganese: 100 mg; vitamin A: 14000 IU; vitamin D3: 2800 IU; vitamin E: 23.80 IU; vitamin K3: 1.96 mg; vitamin B12: 0.025 mg; vitamin B2: 8.4 mg; pantothenic acid: 15 mg; niacin: 1.12 mg; vitamin B6: 4.75 mg; Biotin: 0.10 mg; vitamin B1: 2.50 mg; choline: 1100 mg; folic acid: 50 mg.

**Supplementary Table 2** Primers and their sequences

| **Primer** | **Primer sequence (5’→3’)** |
| --- | --- |
| *C7orf26* | F: CTGAAGGAGATGATGGAGATT; R: GCATTGAGAGGATTGAGTTC |
| *NPAS2* | F: ACAGAGCATCACCAGGAA; R: GTAATAGTCGTAGCCAGAAGT |
| *SERPINF2* | F: GTGGTGGAGATGAAGAGTC; R: CTGATGTGGAGATGGAAGG |
| *PLACL2* | F: CAACGCCAAGCCTATGTG; R: GCAGAAGTCGGAGCAGAT |
| *ISX* | F: GGAAGAGGAAGACCAACTG; R: TGCCAACTGATTGCGAAT |
| *LYGL* | F: GGTTGCTGCTGTTATCTCT; R: CTGCTGTTCCTTACTCCAT |
| *PMLL* | F: CCAGAACCAGACTCTAAGC; R: CTCCAGCACATCCTTGAC |
| *RBBP7* | F: GTCAACTGTCTGTCCTTCA; R: CATCTTCCGCATCCTCTG |
| *CYP2AC2* | F: CAAGAAGAGATAGAGCAAGTG;R: CTGGTGAAGAAGAGGAAGAG |
| *MT4* | F: CCTCAGGACTGCACTTGT; R: CCCTTGGCACAGTTGTTG |
| *CDHR1* | F: CTAAGAGTTCAGGCATCAGA; R: GTATCCAGCAGGCGTATC |
| *MMP13* | F: TTGTGCTTCCTGATGATGAT; R: CCTGTGTCCATAACTTCTGT |
| *CYP2C18* | F: GTTATACCAGATGTTCTCCTAC; R: CTTGTTGTCTCCGTTCCA |
| *MRPS21* | F: AATCACCTCCGCTTCATC; R: CTGCTTCACCGACTCAAT |
| *CCL4* | F: AGTTGTTCTCGCTCTTCTC; R: TGTAGTGCCTCTGGATGA |
| *CCL18* | F: AACCTGCTGCTTGTCCTA; R: CACATATCTCCCTCCCTTTC |
| *CCL26* | F: AACTGCTGGATTCAGATGG; R: TTGCTGCTGGTGATGTAG |
| *XCL1* | F: GGTCATAGTCTGGCTTGG; R: CGTCTTCTGTCTATTCTCTTC |
| *IL-1β* | F: ACATCAACCAGAAGTGCTT; R: GTCCAGGCGGTAGAAGAT |
| *IL-6* | F: CCTCCTCGCCAATCTGAA; R: CCTCACGGTCTTCTCCATA |
| *IL-8L1* | F: GCTCTTCTCCTGATCTCAAT; R: CACACTTCTCTGCCATCTT |
| *IL-8L2* | F: AAGGTAGGACGCTGGTAA; R: CAGTGGTGCATCAGAATTG |
| *IL-34* | F: GCAGTACATGAAGCACAAC; R: CAGGACCTTGAGGCAGTT |
| *LITAF* | F: GAGGAGACAGTAGGAATCAAT; R: CGGTCATAGAACAGCACTA |
| *TNFSF8* | F: TTGTTGCGTTACTCGTCTT; R: CCTGTTGACTTCGTTGTTG |
| *TNFSF10* | F: CCGTCACCTACATCTACTTC; R: GGTCAGCCACTCTGTCTT |
| *TNFSF13B* | F: CTATTGTCAACGCAGAAGAA; R: CGGATAAGACTGTGGCATA |
| *TNFSF15* | F: TTACCAAGAACAACCTGAGT; R: GTGCTCCTTAGTGTAATCCA |
| *IFNG* | F: CTCAAGTCATTCAGATGTAGC; R: TGGATTCTCAAGTCGTTCAT |
| *MX1* | F: CAATCCACGGTCCAACTT; R: GTCCTCTTCTCTGTCATTCA |
| *IRF5* | F: CAACAAGAGCCGTGAGTT; R: CCTTCCGCAGTCATCAATA |
| *IRF7* | F: GCAGCACTGTAACATCTCT; R: GGCACATTCCTCACTCAC |
| *IRF9* | F: ACATCTCCGAGCCTTACA; R: CTCCTTCACCAGCACATC |
| *AvBD10* | F: GTTCTCCTCTTCCTCTTCC; R: AATCTTGGCACAGCAGTT |
| *GAPDH* | F: ACTGTCAAGGCTGAGAACGG; R: AGCTGAGGGAGCTGAGATGA |
| *Chick-P65* | F: ATGGAGCCCGCGGATCTGCTGC;  R: CCTTCATGCCCCTCCAGTTGCCCCC |

Note: “F” and “R” indicate forward and reverse primers, respectively.

**
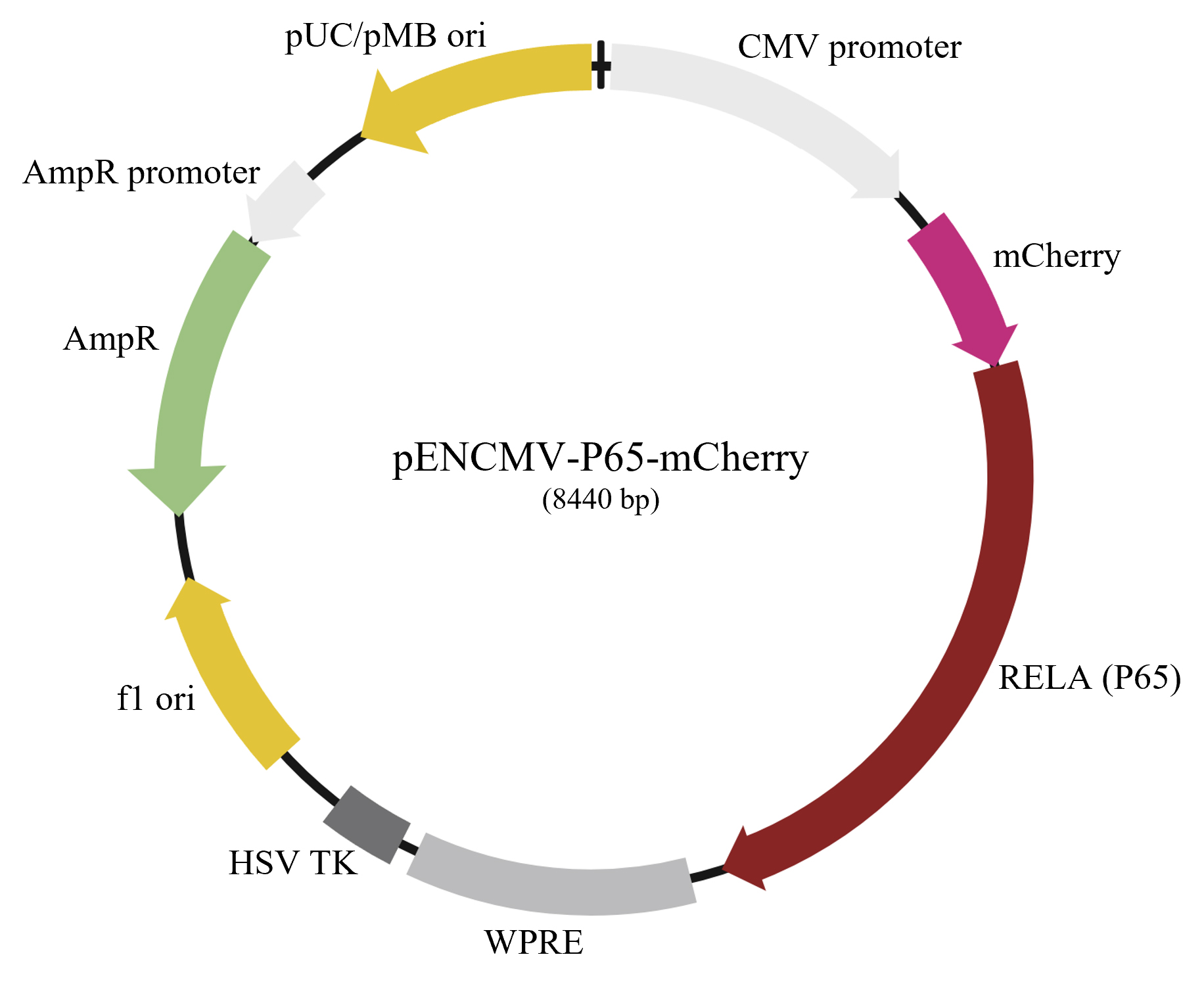
Supplementary Figure 1** Structural map of plasmid pENCMV-P65-mCherry.

**
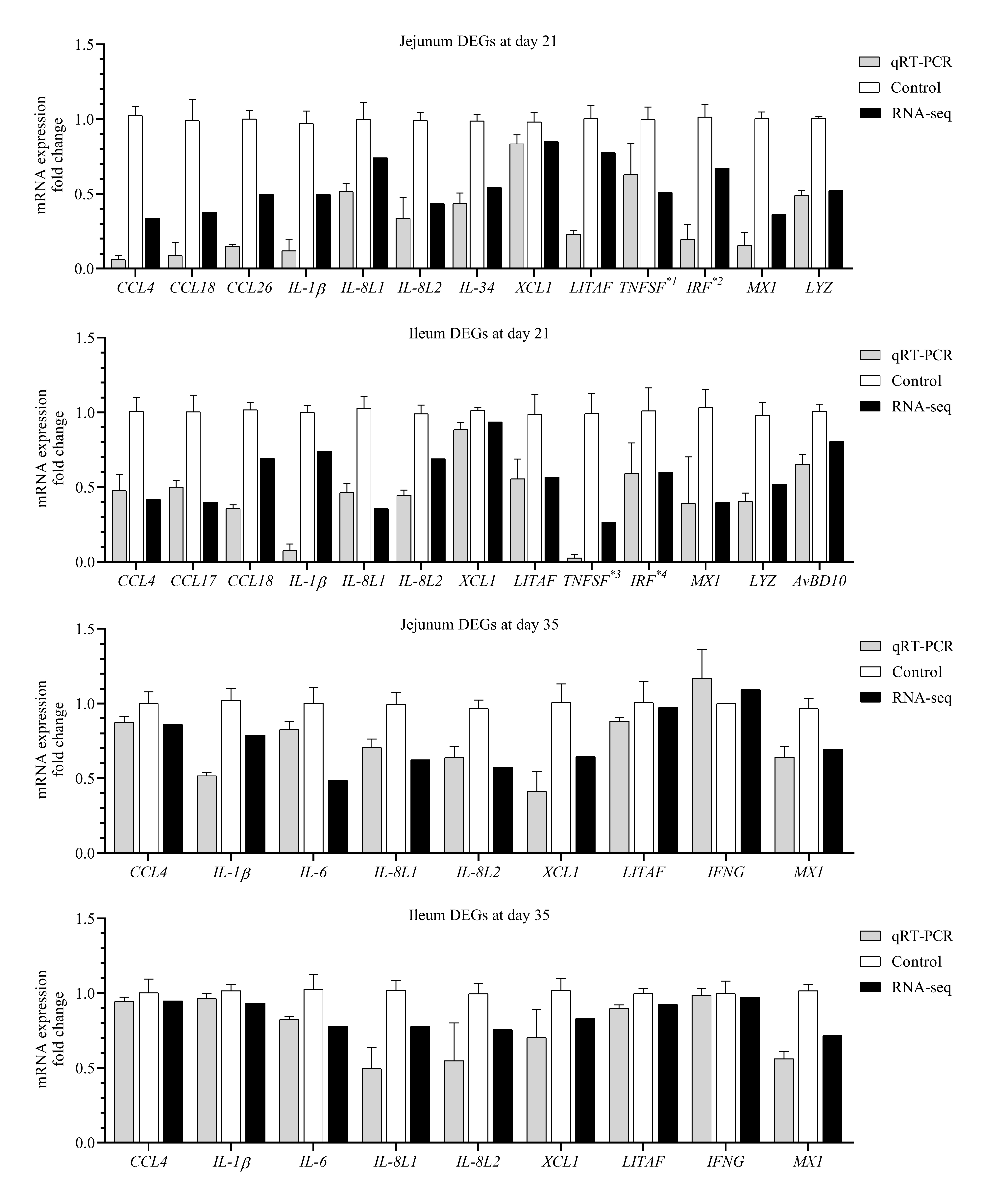
Supplementary Figure 2** Validation of differentially expressed genes (DEGs) from transcriptome sequencing based on qRT-PCR. “^*1^” includes *TNFSF4*, *TNFSF10*, *TNFSF11*, and *TNFSF13*; “^*2^” includes *IFN-γ*, *IRF1*, *IRF5*, and *IRF7*; “^*3^” includes *TNFSF8*, *TNFSF10*, and *TNFSF15*; “^*4^” includes *IFN-γ*, *IRF1*, *IRF2*, *IRF5*, *IRF6*, *IRF7*, and *IRF9*.

**
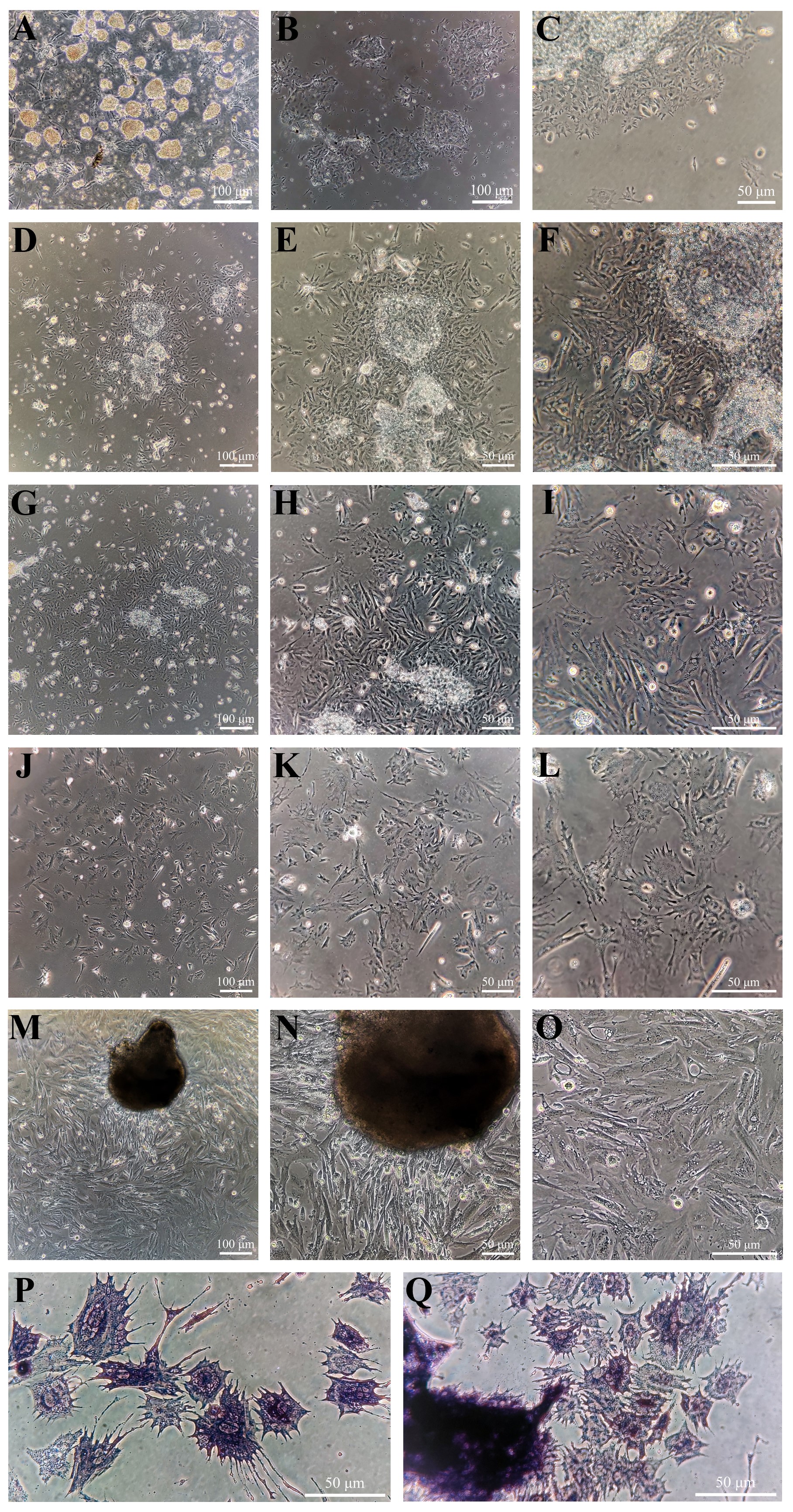
**

**Supplementary Figure 3** Morphological observation and preliminary identification of the primary chicken embryonic small intestinal epithelial cells (PCIECs). **(A)** Morphology of digested free intestinal epithelial crypt cell mass. **(B)** Morphology of newly adherent intestinal crypt cell mass. **(C)** Morphology of intestinal epithelial cells grown by cell mass radiation. **(D–F)** Adherent morphology of PCIECs at 1 d (magnifications of 100×, 200×, and 400×). **(G–I)** Adherent morphology of PCIECs at 3 d (magnifications of 100×, 200×, and 400×). **(J–L)** Adherent morphology of PCIECs at 7 d (magnifications of 100×, 200×, and 400×). **(M–O)** Adherent morphology of PCIECs at 14–21 d (magnifications of 100×, 200×, and 400×). **(P)** Scattered growing PCIECs stained by alkaline phosphatase for 12 h. **(Q)** PCIECs grown from intestinal crypt cell clusters stained by alkaline phosphatase for 12 h.
